# RFX1–dependent activation of SHP-1 induces autophagy by a novel obatoclax derivative in hepatocellular carcinoma cells

**DOI:** 10.18632/oncotarget.2054

**Published:** 2014-06-03

**Authors:** Jung-Chen Su, Ping-Hui Tseng, Cheng-Yi Hsu, Wei-Tien Tai, Jui-Wen Huang, Ching-Huai Ko, Mai-Wei Lin, Chun-Yu Liu, Kuen-Feng Chen, Chung-Wai Shiau

**Affiliations:** ^1^ Institute of Biopharmaceutical Sciences, National Yang-Ming University, Taipei, Taiwan; ^2^ Department of Medical Research, National Taiwan University Hospital, Taipei, Taiwan; ^3^ National Center of Excellence for Clinical Trial and Research, National Taiwan University Hospital, Taipei, Taiwan; ^4^ Division of Hematology and Oncology, Department of Medicine, Taipei Veterans General Hospital, Taipei, Taiwan; ^5^ Biomedical Technology and Device Research Labs, Industrial Technology Research Institute, Hsinchu, Taiwan; ^6^ Institute of Biochemistry and Molecular Biology, National Yang-Ming University, Taipei, Taiwan

**Keywords:** SHP-1, STAT3, autophagy, RFX1, obatoclax derivative

## Abstract

Obatoclax is a small molecule which targets the Bcl-2 family, and is to treat leukemia, lymphoma and lung carcinoma. Previously, an obatoclax analogue, SC-2001, was found to disrupt the protein-protein interactions of the Bcl-2 family and also repress Bcl-XL and Mcl-1 expression via STAT3 inactivation. Here, we report a novel mechanism of autophagy induction by SC-2001 in liver cancer cells. The findings indicate that SC-2001 induced the autophagy marker LC3-II in four hepatocellular carcinoma (HCC) cells. Autophagosomes induced by SC-2001-treated cells were confirmed by electron microscopy. SC-2001 activated SHP-1, dephosphorylated STAT3 and Mcl-1, and subsequently released free beclin 1. Overexpression of STAT3 and Mcl-1 in PLC5 cells attenuated the induction of SC-2001 on autophagy. Abolishment of SHP-1 by a specific inhibitor aboragated the autophagic effects induced by SC-2001. In addition, it was further revealed that RFX-1, a transcription factor of SHP-1, is a critical regulator in SC-2001-mediated autophagy. Downregulation of RFX-1 by si-RNA protected cells from SC-2001-induced autophagy. Importantly, Huh7 tumor-bearing nude mice treated with SC-2001 showed downregulation of Mcl-1 and p-STAT3 protein expression and upregulation of SHP-1, LC3II, and RFX-1 protein expression. In summary, our data suggest that SC-2001 induces autophagic cell death in a RFX1/SHP-1/STAT3/Mcl-1 signaling cascade.

## INTRODUCTION

Autophagy is a cellular catabolic degradation process that results in the autophagosomic-lysosomal degradation of cytosolic proteins and other cellular components [1]. The first step of autophagy is the initiation of vesicle nucleation followed by the formation of an autophagosome. Lipidated LC3-II isoform is a well-known autophagy marker, and it appears in both inner and outer membrane of autophagosome during the nucleation and expansion of vesicle. The second step is a docking and fusion process in which the autophagolysosome is formed by the interaction of autophagosome, endosomes, as well as the lysosomes. Biomolecules embedded in the autophagolysosome are degraded by acid-dependent enzymes into metabolic fuel [2]. Under stressed conditions, both cell survival or cell death can mediated by autophagy, therefore, the fate of cells is dependent on the type of cellular stress [3-5].

Beclin 1 (also called Atg6) is essential for double-membrane autophagosome formation, which is a vital step in autophagic nucleation [6]. Anti-apoptotic Bcl-2 homologues bind to beclin 1 and negatively regulate the autophagy induction [7]. For example, under normal conditions Mcl-1 and beclin 1 form a complex and prevent beclin 1 along from forming a double-membrane autophagosome. Therefore, either downregulation of Mcl-1 or the disruption of the Mcl-1–beclin 1 interaction would be a strategy to induce autophagy [8]. In addition, by chemical disruption of beclin 1 with other anti-apoptotic proteins, such as Bcl-2, and Bcl-XL, free beclin 1 from the beclin 1/Bcl-2 complex can activate autophagic effect [9, 10]. For instance, ABT737, one BH3-mimetic compound, prevents beclin 1 from interacting with Bcl-2/Bcl-XL complex then further induces autophagy [11]. Small interfering RNA screening has showed that several mTORC1 independent signaling cascades are related to autophagy, including STAT3, MAPK, CXCR4 [12]. In addition, recent studies have shown that the activation of autophagy induced by anti-cancer agents, such as sorafenib [13], temsirolimus [14], vorinostat [15] and obatoclax [16] is via a novel mechanism.

Obatoclax is a BH3 mimetic that can interact with BH3 domain belonged to anti-apoptotic Bcl-2 family then blocks the function of anti-apoptotic proteins and release pro-apoptotic proteins. In preclinical studies, obatoclax showed potent anti-tumor activity predominantly in leukemia and myeloma cells [17, 18]. The effect of obatoclax-induced apoptosis in lung cancer [19, 20] and breast cancer [21], is not as potent as that seen in leukemia. However, obatoclax has been suggested to have a synergistic effect when used with lapatinib, a targeted therapy for ErbB2-positive breast cancer, to induce apoptotic and autophagic cell death in human breast and colon cancer cells [22, 23].

We previously demonstrated that SC-2001, a novel obatoclax derivative, shows a better apoptotic efficacy than obatoclax in hepatocellular carcinoma cells [24]. SC-2001 not only inhibits the function of Bcl-2 family but also regulate represses anti-apoptotic protein expression. We further discovered a novel mechanism by which SC-2001 activates SHP-1 phosphatase and reduces the phosphorylation of STAT3 in HCC cells. SC-2001-mediated STAT3 dephoshphorylation inhibits the transcription activities of STAT3 and expression of its downstream genes which induces HCC cell apoptosis.

In our paper, we revealed that SC-2001 showed more potent effect on inducing of autophagy than the lead compound, obatoclax, and has the different drug target. We proved that SC-2001 can disruption of the beclin 1–Mcl-1 interaction and repressed Mcl-1 expression, resulting in free beclin 1 for the induction of autophagy. Further, we found that SC-2001-induced RFX-1 is crucial to SHP-1 activation and in modulating SC2001-mediated autophagy. Most important, SC-2001-indcued RFX-1/SHP-1-dependent inhibition in HCC cells was demonstrated in vivo in a xenograft mouse model. Overall, our results demonstrate the involvement of the RFX-1/SHP-1/STAT-3 axis in SC-2001-mediated autophagy. These findings suggest a novel molecular mechanism of SC-2001-mediated autophagic cell death and highlight the potential of SC-2001 in the development of RFX-1/SHP-1/STAT-3-targeted anticancer therapeutics.

## RESULTS

### SC-2001 induces autophagic phenomena in HCC cells

**Figure 1:**
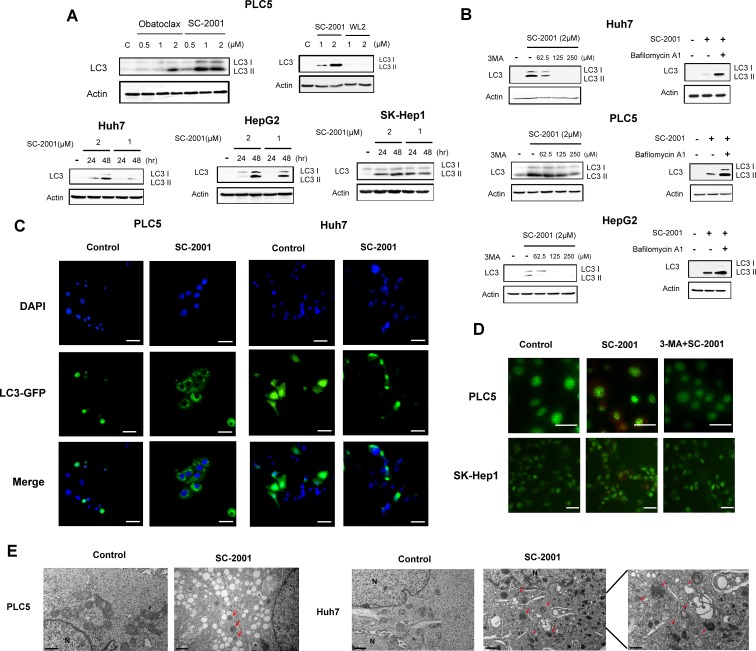
SC-2001-induced autophagy in HCC cells A, SC-2001 induced the generation of LC3-II in a dose- and time-dependent manner. HCC cells were exposed to obatocalx or SC-2001 at various doses for various periods of time. Total lysates were subjected to LC3 protein analysis by Western blot. β-actin was used as a loading control. WL2 was used as a negative control. B, *Left*, autophagy inhibitor, 3-MA, reduced the SC-2001-induced LC3 protein expression in HCC cells. Cells were pretreated with or without 3-MA for 2 h, then treated with 2 μM of SC-2001 for 48 h. Total cell lysates were analyzed by western blot to determine LC3 protein expression. *Right,* SC-2001 activates autophagic flux in HCC cells. Cells were treated with or without 2 μ*M of SC-2001 in the presence of or absence of* bafilomycin for 48 h. Total cell lysates were analyzed by western blot to determine LC3 protein expression. C, PLC5 and Huh7 cells expressing GFP-LC3 were treated with 2 μM of SC-2001 for 48 h. The cells were fixed with paraformaldehyde and visualized with epifluorescence. The punctate pattern of GFP-LC3, representative of autophagosomes, and nuclei were visualized using DAPI staining. Scale bar represents 50 μm. D, PLC5 and SK-Hep-1 cells were treated with 2 μM of SC-2001 alone or SC-2001+3-MA. Images showing acridine orange (AO) staining were taken 48 h post-treatment using fluorescence microscopy. Average numbers of acidic vacuolar organelles (AVOs) per cell were counted in three fields for each condition. Scale bar represents 50 μm. E, Transmission electron microscopy (TEM) images showing autophagosome (arrow) formation in PLC5 and Huh7 cells treated with 2 μM of SC-2001 for 48 h. Scale bar represents 0.5 or 1 μm.

To explore the efficacy of autophagy induction caused by obatoclax and SC-2001, we used HCC cells as model to assess the LC3-II expression level. LC3-II activation represents the autophagic activity. As shown in Figure 1A, treatment with SC-2001 resulted in dose-dependent induction of LC3-II to higher levels than obatoclax in PLC5 cells. WL-2, an obatoclax derivative without any cytotoxicity in HCC cells, was used as a negative control. SC-2001 also exhibited the same effect in Huh7, HepG2 and SK-Hep-1 cells in a time dependent manner. Next, we applied two autophagy inhibitors to verify that the observed autophagic effect was induced by SC-2001. 3-Methyladenine (3-MA), a widely-used inhibitor of the initial phase of the autophagic process prevented LC3 II formation from LC-3-I in the presence of SC-2001. Bafilomycin A1, which inhibits the docking and fusion process in the late stage of autophagy, resulted in significant accumulation of LC3II in cells treated with SC-2001 (Figure 1B). Moreover, the SC-2001-induced autophagic effect was confirmed by GFP-LC3 staining. As shown in Figure 1C, PLC5 and Huh7 cells with GFP-LC3 transfection showed increased GFP-LC3II dots. In addition, autophagy as a result of SC-2001 treatment was confirmed with acridine orange staining. Conversely, 3-MA reduced acidic vesicular organelles in SC-2001-treated cells. (Figure 1D). Finally, the ability of inducing autophagy by SC-2001 was verified by electron microscopy which revealed the formation of autophagosomes in PLC 5 and Huh7 cells (Figure 1E).

### Disruption of beclin 1–Mcl-1 complex interaction and ATG5 expression are associated with SC-2001-induced autophagy

**Figure 2:**
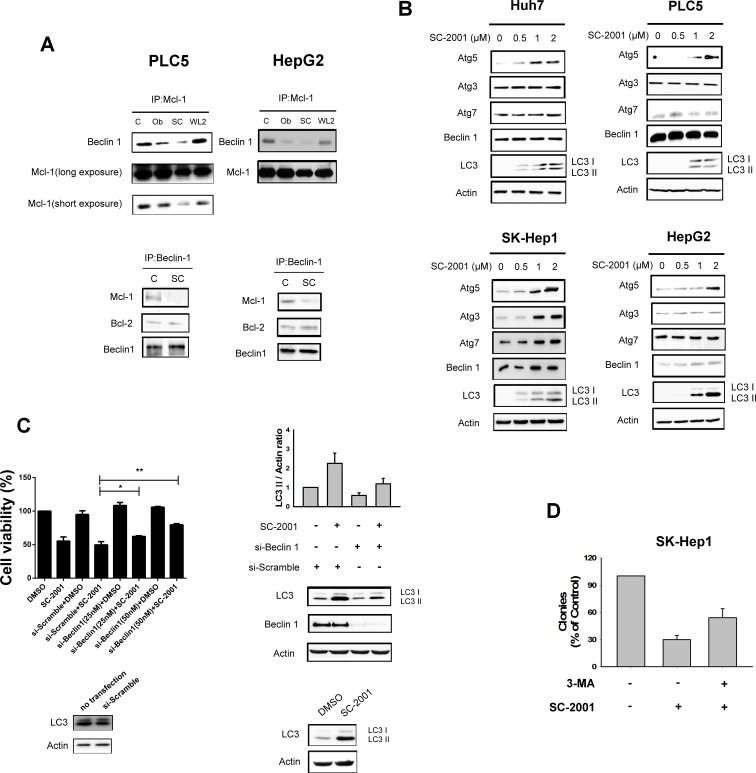
SC-2001 induces autophagic cell death and disrupts the MCL-1beclin 1 complex A, SC-2001 induced the disassociation of beclin 1 and Mcl-1. *Upper,* Mcl-1 was immunoprecipitated from PLC5 or HepG2 cells treated with 2 μM of SC-2001 or obatocax for 48 h and analyzed for the presence of beclin 1. WL-2 was used as a negative control. *Lower,* Beclin 1 was immunoprecipitated from PLC5 or HepG2 cells treated with 2 μM of SC-2001 for 48 h and analyzed for the presence of Mcl-1 and Bcl-2. B, Effects of SC-2001 on autophagy-related proteins in HCC cells. Cells were treated with SC-2001 at the indicated dose for 48 h. C, PLC5 cells were transfected with control siRNA or beclin 1 siRNA for 48 h then treated with SC-2001 at 2 μM for 48 h. *Left*, silencing beclin 1 by siRNA reduces the effects of SC-2001 on cell proliferation in HCC cells. The off-target effect of the si-RNA can be ruled out by comparison between the group of no-transfection and si-scramble. *Right*, silencing beclin 1 by siRNA reduces the effects of SC-2001 on LC3II protein expression. The off-target effect of the si-RNA can be ruled out by no-transfection group. D, SC-2001 treatment inhibits clonogenic survival of SK-Hep1 cells. 3-MA rescued SK-Hep1 cells from autophagic cell death induced by SC-2001 treatment. Cells were treated with SC-2001 for 48 h, and clonogenic survival in crystal violet was assessed after incubation for two weeks.

Our previous report showed that inhibition of STAT-3 and downregulation Mcl-1 are key factors in SC-2001-induced cell apoptosis [24]. To elucidate the biological and pharmacological effect of SC-2001-induced autophagy, the ability of SC-2001-induced disruption of the Mcl-1–beclin 1(ATG6) complex and/or other ATG family members were tested in HCC cells. SC-2001 disrupted Mcl-1 and beclin 1 interaction and further released beclin 1 to initiate autophagy, but did not influence the complex of beclin 1 and Bcl-2 (Figure 2A). Furthermore, the expression of ATG5 was increased by SC-2001 in concentrate-dependent fashion in HCC cells. On the other hand, SC-2001 showed no influence on ATG3, ATG7 or beclin 1 in HCC cells (Figure 2B). Genetic knockdown of beclin 1 by siRNA reversed LC3-II production and partially rescued cell viability (Figure 2C). Also, 3-MA reversed the reduction of SC-2001 induced-colony formation (Figure 2D). These data suggest that disruption the interaction between beclin 1 and Mcl-1 and induction of ATG5 expression is vital to SC-2001-induced autophagy.

### SC-2001-induced autophagic effect via a SHP-1/STAT3/Mcl-1-dependent signaling cascade

**Figure 3:**
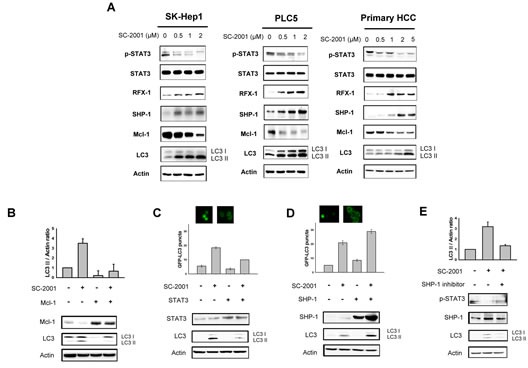
SHP-1-STAT3 signaling is involved in SC-2001-induced autophagy A, SK-Hep-1 and PLC5 cells were treated with SC-2001 for 48 h. Total cell lysate of SK-Hep-1 and PLC5 were subjected to pSTAT3, SHP-1, STAT3, Mcl-1, and LC3 protein analysis by Western blot. β-actin was used as a loading control. B, PLC5 cells were transfected with Mcl-1 overexpression plasmid. After transfection for 48 h, cells were treated with 2 μM of SC-2001 for 48 h. Total cell lysates were subjected to Mcl-1 and LC3 protein analysis by Western blot technique. β-actin was used as a loading control. C, PLC5 cells were transfected with STAT3 overexpression plasmid and stable clones were selected by G418. Cells were treated with 2 μM of SC-2001 for 48 h. Total cell lysates were subjected to STAT3 and LC3 protein analysis by Western blot. β-actin was used as a loading control. D, PLC5 cells were transfected with SHP-1 overexpression plasmid and stable clones were selected by G418. Cells were treated with 2 μM of SC-2001 for 48 h. Total cell lysates were subjected to SHP-1 and LC3 protein analysis by Western blot. β-actin was used as a loading control. E, PLC5 cells were treated with SHP-1 inhibitor. After treatment for 2 h, cells were treated with 2 μM of SC-2001 for 48 h. Total cell lysates were subjected to pSTAT3, SHP-1 and LC3 protein analysis by Western blot. β-actin was used as a loading control. Each band of LC3II was normalized to their actin after quantification by densitometer.

Our previous study showed that SHP-1 expression is critical to SC-2001-indcued apoptosis [24]. To further examine the involvement of SHP-1 induced by SC-2001 in autophagy in HCC, we verified the SHP-1/STAT3/Mcl-1 pathway. SC-2001 significantly induced SHP-1 expression and reduced the status of phoshpho-STAT3 and Mcl-1 expression in a concentrate-dependent fashion in SK-Hep-1 and PLC5 and primary HCC cells (Figure 3A). These results correlated with LC3-II production. Moreover, overexpression of STAT3 and transiently overexpressed Mcl-1 in PLC5 cells abolished SC-2001-induced LC3II production (Figure 3B and 3C). Enhanced SHP-1 expression synergized the effect of SC-2001 treatment on LC3-II expression (Figure 3D). In addition, an SHP-1 inhibitor compromises the effect of SC-2001 on LC3-II (Figure 3E). Taken together, we conclude that SHP-1/STAT3/Mcl-1 is important in SC-2001-induced autophagy.

### RFX-1 regulates SC-2001-mediated SHP1 transcription in HCC cells

**Figure 4:**
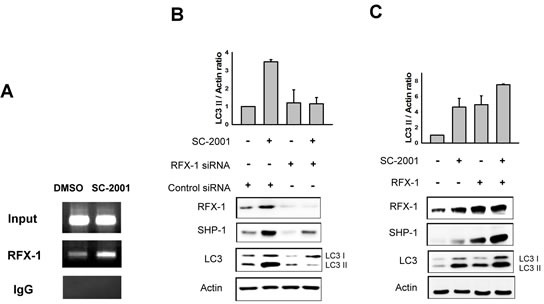
RFX-1 expression is related to LC3II protein level A, PLC5 cells were treated with 2 uM of SC-2001 for 48 h. DNA from control and SC-2001-treated PLC5 cells was immunoprecipitated with RFX-1 or rabbit IgG antibody and captured by protein A-agarose beads. RFX-1 binding site fragment in SHP-1 promoter was detected by PCR in CHIP samples. B, PLC5 cells were transfected with RFX-1 si-RNA for 48 h and treated with 2 μM of SC-2001 for 48 h. Total lysates of control and SC-2001-treated groups were subjected to RFX-1, SHP-1 and LC3 protein analysis by Western blot. β-actin was used as a loading control. C, PLC5 cells were transfected with RFX-1 overexpression plasmid for 48 h and treated with 2 μM of SC-2001 for 48 h. Total lysates of control and SC-2001-treated groups were subjected to RFX-1, SHP-1 and LC3 protein analysis by Western blot. β-actin was used as a loading control. Each band of LC3II was normalized to their actin after quantification by densitometer.

According to the literature, RFX-1 is an important transcription factor of SHP-1 [25]. To investigate whether RFX-1 is involved in SC-2001-induced SHP-1 expression and autophagy, we examined the expression level of RFX-1 in HCC cells with the treatment of SC-2001. As shown in Fig 3A, RFX-1 was upregulated by SC-2001 in a concentration-dependent fashion. Next, to examine the involvement of RFX-1 in SC-2001-activated SHP-1 transcription, we applied chromatin IP to demonstrate binding capacity. As shown in Figure 4A, the binding activity of RFX-1 to the SHP-1 promoter was obviously increased. Furthermore, RFX-1 silencing by si-RNA abolished SC-2001 efficacy on SHP-1 and LC3II expression (Figure 4B). In addition, we verified the effect of the combination of SC-2001 and ectopic expression of RFX-1 in PLC5 cells. The results demonstrated that treatment of RFX-1 overexpressed cells with SC-2001 significantly increased autophagy (Figure 4C).

### SC-2001 inhibits tumor growth via RFX-1/SHP-1/STAT3-dependent activation of autophagy

**Figure 5:**
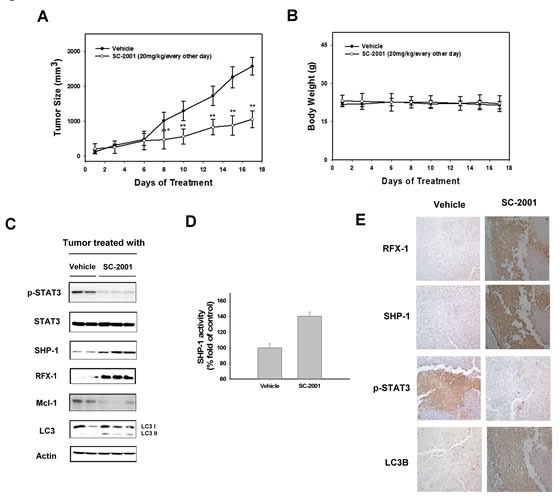
SC-2001 inhibits tumor growth via RFX-1/SHP-1/STAT3 dependent autophagic cell death A, Huh7 tumor-bearing mice were treated with or without SC-2001 at 20 mg/kg orally every other day. Tumor size was measured and mice were sacrificed after 21 days. B, Body weights of control and SC-2001-treated mice were measured. C, Western blot analysis of p-STAT3, STAT3, SHP-1, RFX-1, Mcl-1 and LC3 in Huh7 tumors. D, SHP-1 activity in control and SC-2001-treated Huh7 tumors. E, Immunohistochemistry of RFX-1, SHP-1, p-STAT3, and LC3B in control and SC-2001-treated Huh7 tumors.

To explore whether SC-2001's pharmacological effect on autophagy may be therapeutically relevant, the *in vivo* consequences of SC-2001 were examined. Mice bearing Huh7 tumors randomized to treatment groups and treated with vehicle or SC-2001 (20 mg/kg/every other day). SC-2001-treatment groups exhibited significantly tumor growth inhibition effect (Figure 5A). All animals tolerated SC-2001 treatment well. The mice did not show any visible signs of toxicity or body weight loss (Figure 5B). To confirm the biological mechanism identified in vitro, we next examined the efficacy that SC-2001-induced autophagic effect in HCC tumors. SC-2001 treatment increased RFX-1 and SHP-1 levels and obviously induced LC3-II (Figure 5C). SC-2001 treatment also activates the SHP-1 activity in Huh7 tumors (Figure 5D). In addition, negative regulators of autophagy including p-STAT3 and Mcl-1 were decreased. Also, IHC staining proved the effect of SC-2001 on autophagy (Figure 5E). We have now demonstrated that SC-2001 considerably inhibits tumor growth *in vivo*. Equally important, we discovered that SC-2001 activated a novel RFX-1/SHP-1 autophagic regulation in HCC.

## DISCUSSION

SC-2001 was originally derived from obatoclax as a Bcl-2 family inhibitor against human cancers [26]. In our previous study [24], SC-2001 was identified to be SHP-1 agonist, and a negative regulator of STAT3 (which is over-expressed in various cancers), by enhancing SHP-1 expression via transcription activity. SC-2001 blocks STAT3 phosphorylation activity, a process required for survival and proliferation signals, and inactivates STAT3 transcription. Subsequent reduction of several regulatory substrates of STAT3, such as cyclin D and survivin, leads to inhibition of tumor growth mainly through induction of apoptosis. Whether SC-2001 is involved in other types of cell death in cancer cells, such as autophagy, was totally unknown. In our paper, we demonstrated that SC-2001 is a potent autophagy-inducing agent through activation of the RFX-1/SHP-1/STAT3 axis in HCC cells.

Autophagy is a cellular catabolic degradation process that is triggered in response to cellular stress. Autophagy is separated from four major processes, initiation, nucleation, cycling, and expansion, which involve several key genes [27]. For example, Atg1 joints with Atg13 and FIP200 in autophagy initiation. In addition, complexs formed by beclin 1, class III (Vps34) PI3Ks, and UVRAG are crucial for the autophagic process [28]. Through acting jointly with beclin 1, the pro-survival Bcl-2 family proteins, including Bcl-2, Bcl-xl and Mcl-1 abolish autophagy initiation [10, 29, 30]. These Bcl-2 family members disrupt the beclin/Vsp34/UVRAG complex by competitive binding with beclin 1 and inhibit autophagy. Alternatively, suppression of pro-survival Bcl-2 family members may promote autophagy [31]. Obatoclax prevents beclin 1 from interacting with protective Bcl-2 family members, leading to release the free beclin/Vsp34/UVRAG complex and induction of autophagy.

Here, we investigated the efficacy of SC-2001 and the mechanism by which it activates autophagic effect in HCC cells. First, by measuring LC3-I to LC3-II transition, we found that SC-2001 induces autophagic effect in both time- and concentration-dependent fashion. SC-2001 induced higher levels of autophagy than obatoclax. SC-2001-induced autophagosome formation was verified by electron microscopy. Moreover, after transfection of GFP-LC3 into PLC5 and Huh7 cells, treatment with SC-2001 caused GFP-LC3 puncta formation reflecting the progression of autophagic flux. In agreement with this result, acridine orange-stained autophagic vesicles were shown after SC-2001 treatment in PLC5 as well as SK-Hep-1 cells. These results demonstrated that SC-2001 can induce autophagy in HCC cells.

Previously, we demonstrated that SC-2001 downregulates Mcl-1 through the SHP-1/STAT3 pathway. Therefore, we hypothesized that SHP-1 might play an important role in autophagy. A SHP-1 specific inhibitor blocked SC-2001-induced LC3-II expression in HCC cells. Conversely, ectopic expression of SHP-1 enhanced the LC3-II expression under SC-2001 treatment in HCC cells, indicating the involvement of SHP-1 in autophagy. Besides the demonstration that SHP-1 modulates SC-2001-activated autophagy, our results suggest RFX-1 to be the key regulator in SC-2001-induced autophagy upstream of the SHP-1/STAT3/Mcl-1 axis. Ectopic expression of RFX-1 leads to subsequently enhanced effects of SC-2001 on autophagy, implying its crucial role in SC-2001-induced autophagic phenomenon. Conversely, RFX-1 inhibition by siRNA resulted in a reduction of SC-2001-induced LC3-II. In a nude mouse xenograft model, SC-2001 repressed tumor growth and the expression level of RFX-1 and SHP-1, and LC3-II was significantly increased in tumor cells, indicating SC-2001-induced autophagic cell death.

It should be noted that in many instances autophagy constitutes a cytoprotective response activated by dying cells in the attempt to cope with stress, and its inhibition accelerates cell death [32]. In this regards, inhibition of autophagy may enhance the efficacy of currently used antineoplastic agents in cancer cells and has been suggested as a potential anti-cancer strategy [33-35]. On the other hand, several studies have shown autophagy may, in some circumstances, mediates the cytotoxic or cytostatic effect of anticancer agents, in which blockade of autophagy abolishes the therapeutic actions [36, 37]. For example, Turcotte et al [38] reported a novel compound STF-62247 induced autophagic cell death in renal cell carcinoma cells. Interestingly, it has been reported that autophagy mediated the anti-cancer effect of a natural BH3-memimtic, (–)-gossypol, in androgen-resistant prostate cancer cells [39] and in malignant glioma cells [40]. It appears that autophagy plays a paradoxical role in cell-fate decision mechanisms [36, 37]. Based on the new, revised Nomenclature Committee of Cell Death (NCCD) classification on cell death in 2012 [32], Galluzzi *et al* reintroduced the term ‘autophagic cell death’ to indicate a cell death that is mediated by autophagy and can be suppressed by the inhibition of the autophagic pathway. On the basis of the NCCD 2012 definition, all cases of cell death that exhibit markers of autophagy but cannot be blocked by autophagy inhibition (such as RNAi targeting beclin 1, or inhibitors of Vps34, a class III phosphoinositide 3-kinase (PI3-kinase) should not be classified as autophagic cell death [32]. Accordingly, our current study showed that SC-2001-induced autophagy in association with cytotoxic/cytostatic effects can be suppressed by Beclin 1 (Figure 2C) or by 3-MA, a broad-spectrum PI3-kinase inhibitor (Figure 1B, 1D, and 2D). In addition, we have added supplementary data showing SC-2001 promotes cancer cell death (Supplementary figure 1). Our current data suggest autophagy mediates part of the anti-cancer effects of SC-2001 in HCC cells. Moreover, our previous study has shown that SC-2001 induces apoptosis by modulating SHP-1/pSTAT3 mechanism in HCC cells [24]. Collectively, we suggest that SC-2001 not only induces apoptosis, but is also able to induce autophagic cell death. We have added a comprehensive plot to provide current known drug mechanisms of SC-2001 (Supplementary figure 2).

We conclude that SC-2001 induces autophagy in novel RFX-1/SHP-1/STAT3/MCL-1 mechanism: RFX-1-dependent Mcl-1 downregulation. This study identifies RFX-1 as a major mediator of SC-2001-induced autophagy and indicates that RFX-1 may play a tumor suppressor role in HCC. Modification of SC-2001 as a RFX-1 agonist might have clinical effect in the treatment of human HCC.

## MATERIALS AND METHODS

### Reagents and antibodies

Bafliomycin A1 (Sigma-Aldrich, B1793) and 3-MA (Sigma-Aldrich, M9281) were purchased from Sigma. SHP-1 inhibitor (Calbiochem, 540210) was purchased from Merk. Antibodies for immunoblotting were: SHP-1 (Abcam, ab32559), Bcl-xl (Abcam, ab32310), p-STAT3-Tyr705 (Cell Signaling, #7300), STAT3 (Cell Signaling, #4904), LC3 (Cell Signaling, #3868), Mcl-1 (Cell Signaling, #4572), beclin 1 (Cell Signaling, #3495), Atg5 (Cell Signaling, #2630), Atg3 (Cell Signaling, #3451), Atg7 (Cell Signaling, #2631), RFX-1 (Novus Biologicals, NBP1-52652).

### Cell Culture

The Huh-7 HCC cell line was obtained from the Health Science Research Resources Bank (HSRRB, JCRB0403). The PLC/PRF/5 (PLC5), HepG2 and SK-Hep1 cell lines were obtained from American Type Culture Collection (ATCC). All cells obtained from HSRRB or ATCC were immediately expanded and frozen such that all cell lines could be restarted every 3 months from a frozen vial of the same batch of cells. No further authentication was conducted in our laboratory. Cells were maintained in DMEM (Sigma-Aldrich, D5648) supplemented with 10% fetal bovine serum (FBS, Hyclone, SH30071.03), 100 units/mL penicillin G and 100 μg/mL streptomycin solution (Sigma-Aldrich, P4333) in a humidified incubator at 37°C in an atmosphere of 5% CO_2_ in air. Primary cancer cells from consenting patients were also analyzed. Study protocols were approved by the institutional review board of the institution, and informed consent was obtained in accordance with the Declaration of Helsinki. Human HCC samples were obtained from the patient who underwent tumor resection. The cells were isolated by mechanical mincing and digestion by collagenase.

### Gene knockdown using siRNA

Smart-pool small interfering RNAs (siRNAs), including the control (D-001810-10), SHP-1 (Dharmacon, L-009778-00-0005), RFX-1 (Dharmacon, L-010147-00-0005), beclin 1 (Dharmacon, L-010552-00-0005) were purchased from Dharmacon. The knockdown procedure was as described previously [41]. Briefly, PLC5 cells were transfected with siRNAs against the phosphatases given above or the control sequence for 48 hours and then treated with SC-2001 at the indicated concentrations. The cell extracts were analyzed by western blot.

### PLC5 cells with ectopic expression of STAT3, Mcl-1 and RFX-1

STAT3 cDNA was purchased from Origene (Origene, RC215836). STAT3-overexpresed PLC5 cells derived from a single stable clone were prepared for evaluating the major target of SC-2001. Briefly, following transfection, cells were cultured in the presence of 0.8 mg/mL G418 (Gibco, Invitrogen, 11811-031) according to previous reports [42]. After 8 weeks of selection, surviving colonies, i.e., those arising from stably transfected cells were selected and individually amplified. Mcl-1 (Addgene, 25375) or RFX-1 (Genecoeopia, EX-P0104-M29) were transiently overexpressed by transfection technique in PLC5 cells, respectively.

### SHP-1 phosphatase activity

A RediPlate 96 EnzChek Tyrosine Phosphatase Assay Kit (Molecular probe, R-22067) was used for SHP-1 activity assay. The method was as described previously [43].

### Colony formation assay

SK-Hep1 cells were seeded in 6-well plates (~1000-5000 cells per well) and subjected to the indicated treatments, with the drug being removed to terminate the treatment. Two weeks later, plates were washed in PBS, fixed with 100% methanol (Sigma-Aldrich, E7023) and stained with a filtered solution of 5% w/v crystal violet (crystal violet, Sigma-Aldrich, C3886). After washing with tap water, the colonies were counted both manually (by eye) and digitally using a ColCount automated colony counter.

### Autophagy analysis

The following methods were used to assess drug-induced autophagic cell death: (1) Western blot analysis of microtubule-associated protein 1 light chain 3 (LC3 II) as described previously [2, 44]; and (2) electron microscopy. Samples were fixed with 2.5% glutaraldehyde solution buffer in PBS at 4°C for 1 h, postfixed in 1% osmium tetroxide solution at 4°C for 3 h, dehydrated in graded concentrations of ethanol and embedded in LR white resin (Sigma-Aldrich, 62661). Ultrathin sections (70 nm) were examined with a JEOL JEM-1400EX electron microscope at 120 Kv. Representative areas were recorded at 20,000, 40,000 magnification; (3) Immunofluorescence of acridine orange (Sigma-Aldrich, A6014). HCC cells were grown on coverslips. After being washed with PBS, cells were treated with 2 μM SC-2001 or SC-2001+3-MA for 48 h, fixed with ice-cold 4% paraformaldehyde (Sigma-Aldrich, P6148) for 30 minutes at room temperature then stained with acridine orange (5 μg/ml) for 5 minutes at room temperature. Cells were examined under a Leica DM2500 fluorescence microscope.

### Chromatin Immunoprecipitation (CHIP) Assay

The CHIP assay was performed according to the EZ ChIP chromatin immunoprecipitation (Merck Millipore, 17-371) and EZ-Zyme Chromatin Prep Kit (Merck Millipore, 17-375) manufacturer's protocol. Briefly, after cross-linking with 17.5% paraformaldehyde, PLC5 cells were washed with phosphate-buffered saline and lysed in lysis buffer. The DNA was fragmented to about 200–500 base pairs by the EZ-Zyme. Approximately 5 × 10^6^ cells were used per CHIP assay and the resulting DNA fragments were incubated with 2 μg RFX1 antibodies, which were generated from rabbit, or nonspecific rabbit IgG (Cell signaling, #3900). The immunoprecipitated products were washed sequentially with low-salt immune complex wash buffer, high-salt immune complex wash buffer, LiCl immune complex wash buffer, and twice with TE buffer. The chromatin was eluted from the agarose by incubating with elution buffer (1% SDS, 100mM NaHCO_3_); and the DNA–protein complexes were reversely cross-linked by high-salt solution containing 200 mM NaCl at 65°C for at least 5 h. To eliminate contaminations of proteins and RNAs, the mixture was treated with 10 mg RNase A at 37°C for 30 min and then treated with protease K for 2 h at 45°C. Finally, the precipitated DNA was recovered using the spin column provided in the ChIP kit, and eluted with 50 ml elution buffer. PCR reaction was conducted using Taq DNA polymerase (Invitrogen, 4398881). Two microliters of the precipitated DNA was used as template. The sequence of the primers used in the ChIP assay were as follows: 5’-CCTCTTGCAGGTGTCCTTAAG-3’, and 5’-TGGAAAGGCAGAGGGAATCAG-3’.

### TUNEL assay

The TUNEL assay is performed according to the manufacture's instruction (AAT Bioquest, 22844). Briefly, cells were seeded into 96-well plate. After cells were attached to the plate, cells were treated with SC-2001 as indicated concentration for 48 h. After that, cells were fixed with 4% formaldehyde for 20 minutes at room temperature and then washed with PBS. Each well was incubated with TUNEL reaction buffer provided by commercial kit for 1 h at 37°C. The fluorescence intensity was measured by fluorescence microplate reader at Ex/Em = 550/590 nm after incubation.

### Xenograft tumor growth

Male NCr athymic nude mice (5-7 weeks of age) were obtained from the National Laboratory Animal Center (Taipei, Taiwan). All experimental procedures using these mice were performed in accordance with protocols approved by National Taiwan University. When Huh7 tumors reached 150 mm^3^, mice received SC-2001 (20 mg/kg) orally (every other day). Controls received vehicle.

### Statistical analysis

Data are expressed as mean ± SD or SE. Statistical comparisons were based on nonparametric tests and statistical significance was defined at *P* < 0.05. All statistical analyses were performed using SPSS for Windows version 12.0 software.

## SUPPLEMENTARY MATERIALS AND FIGURES


