# Supplementary material for: RFX1–dependent activation of SHP-1 induces autophagy by a novel obatoclax derivative in hepatocellular carcinoma cells
Source: Oncotarget. 2014 Jun 3;5(13):4909–19. doi: 10.18632/oncotarget.2054 (PMC4148109; doi:10.18632/oncotarget.2054)
Supplement: Supplementary file 1 [file oncotarget-05-4909-s001.pdf]

RFX1–dependent activation of SHP-1 induces autophagy by a novel obatoclax derivative in Hepatocellular carcinoma cells

Supplementary Material

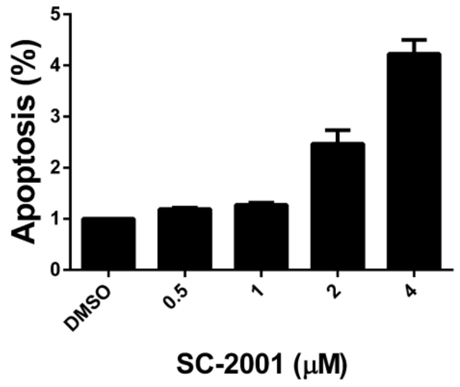

Supplementary figure 1: SC-2001 induced apoptosis of PLC5 cells. PLC5 cells were exposed to different concentration of SC-2001 for 48 h. After that, TUNEL assay were subjected to analyze the apoptotic effect of SC-2001.

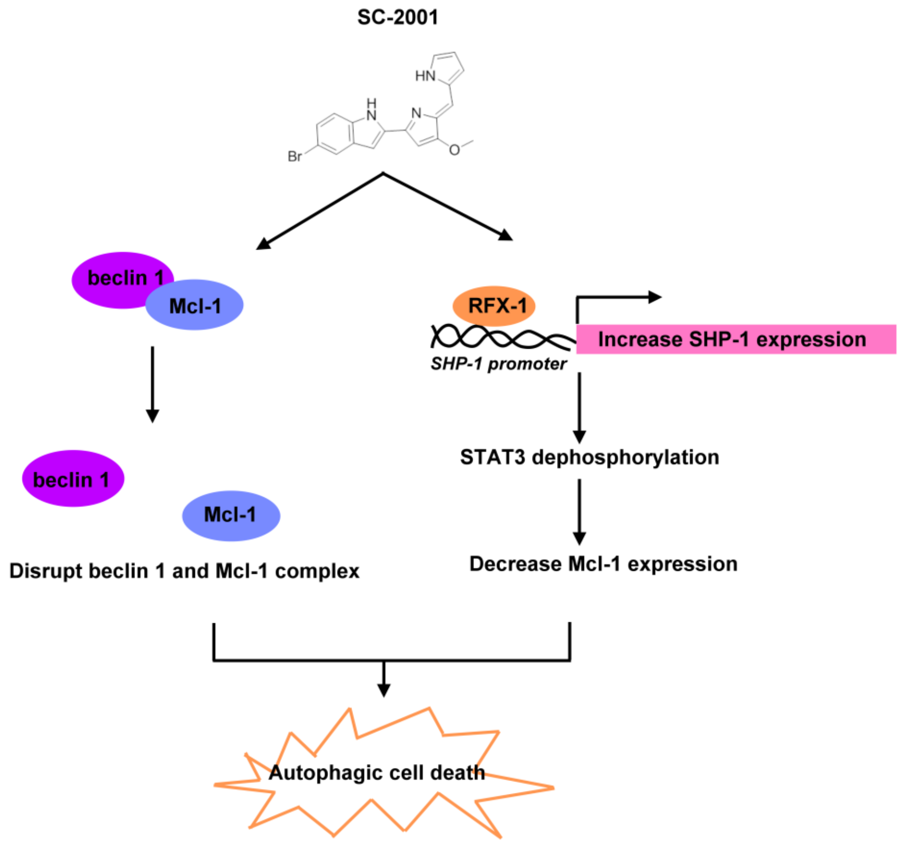

Supplementary figure 2: The model of SC-2001-induced autophagic cell death.
